# Reparation of nano-FeS by ultrasonic precipitation for treatment of acidic chromium-containing wastewater

**DOI:** 10.1038/s41598-023-50070-y

**Published:** 2024-01-02

**Authors:** Mengjia Dai, Junzhen Di, Ting Zhang, Tuoda Li, Yanrong Dong, Sihang Bao, Saiou Fu

**Affiliations:** 1 College of Mining, Liaoning Technical University, Fuxin, 123000 China; 2 College of Civil Engineering, Liaoning Technical University, Fuxin, 123000 China

**Keywords:** Environmental chemistry, Environmental sciences, Nanoscience and technology

## Abstract

Nano-FeS is prone to agglomeration in the treatment of chromium-containing wastewater, and ultrasonic precipitation was used to synthesize nano-FeS to increase its dispersion. The optimization of the preparation method was carried out by single factor method (reaction temperature, Fe/S molar ratio and FeSO_4_ dropping flow rate) and response surface methodology. Dynamic experiments were constructed to investigate the long-term remediation effect and water column changes of nano-FeS and its solid particles. The changes of the remediation materials before and after the reaction were observed by SEM, and the mechanism of the remediation of chromium-containing wastewater by nano-FeS prepared by ultrasonication was revealed by XRD. The results showed that the reaction temperature of 12 °C, Fe/S molar ratio of 3.5 and FeSO_4_ dropping flow rate of 0.5 mL/s were the best parameters for the preparation of nano-FeS. The nano-FeS has efficient dispersion and well-defined mesoporous structure in the form of needles and whiskers of 40–80 nm. The dynamic experiments showed that the average removal of Cr(VI) and total chromium by nano-FeS and its immobilized particles were 94.97% and 63.51%, 94.93% and 45.76%, respectively. Fe^2+^ and S^2−^ ionized by the FeS nanoparticles rapidly reduced Cr(VI) to Cr(III). Part of S^2−^ may reduce Fe^3+^ to Fe^2+^, forming a small iron cycle that gradually decreases with the ion concentration. Cr(III) and Fe^2+^ form Cr(OH)_3_ and FeOOH, respectively, with the change of aqueous environment. Another part of S^2−^ reacts with Cr(III) to form Cr_2_S_3_ precipitate or is oxidized to singlet sulfur. The FeS nanoparticles change from short rod-shaped to spherical shape. Compared with the conventional chemical precipitation method, the method used in this study is simple, low cost, small particle size and high removal rate per unit.

## Introduction

Due to the rapid industrial development and non-ferrous metal mining in recent years, a large amount of acidic wastewater containing pH between 4 and 6 and as low as 2 has been generated^1–4^. This untreated waste has a high concentration of non-biodegradable or naturally attenuated heavy metals in natural water bodies^5,6^. Heavy metal Cr, in specific, is a major contaminant in acidic industrial wastewater^7^. Cr exists in water as Cr(VI) and Cr(VII) (III). Cr(VI) is highly soluble and mobile, and its toxicity is 100 times greater than that of Cr(III)^8,9^. Cr(VI). It has been proven through statistics obtained from the years 2016 to 2018 that Cr(VI) in some national water quality monitoring points surpassed the standard^10^. This exceeding value of a contaminant has seriously threatened not only human health but also had a lasting impact on flora and fauna^11^. Consequently, there is a dire need for the treatment of wastewater which been polluted with acidic chromium. Different methods like the ion exchange method, an adsorption method, a chemical precipitation method, a membrane separation method, and biological treatment methods are presently used for the treatment of chromium-containing wastewater^12,13^. Conventional methods are not applicable for the treatment of wastewater that has been contaminated with chromium due to its high toxicity. At present, the most frequently used treatment method for acidic chromium-containing wastewater is chemical precipitation. In chemical precipitation, alkaline or sulfide salts are added to acidic chromium-containing wastewater for the reduction of Cr(VI) to Cr(III). This results in the formation of precipitates for the removal of Cr(VI) in water^14,15^. As a result, the development of an efficient, cost-effective, and environmentally friendly reducing agent material has become a major technical constraint in the chemical precipitation treatment of acidic chromium-containing wastewater. With the advancement of nanotechnology and material chemistry in recent years, studies on the treatment of chromium-containing wastewater by chemical precipitation have progressed to a new level. Nano-FeS has a high reducing potential. Fe^2+^ and S^2−^ ionized in acidic wastewater can be used as electron donors, reducing Cr(VI) to Cr(III) and forming the intolerant Cr(III)-Fe(III) complex precipitate. As a result, it has significant benefits and broad research opportunities in the treatment of chromium-containing wastewater.

Many researchers are interested in Nano FeS for the treatment of chromium-containing wastewater because of its significant advantages, such as conquering the limitations of conventional materials. The traditional methods used fewer chemicals and resultantly produce less sludge^16,17^. Chemically synthesized nano-Fes have greater specific surface area and reactivity, resulting in higher reactivity and removal efficiency in reducing and immobilizing heavy metals^17–19^. However, FeS nanoparticles synthesized by typical co-precipitation methods are prone to agglomeration due to high surface energy, large van der Waals forces and high density. In order to improve the utilization efficiency of nano-FeS, the surface properties of nano-FeS need to be modified as well as modified, and the FeS particles are mainly functionally stabilized by the addition of loading materials and coating the surface of bare nano-FeS particles with polymers or surfactants^20–23^. Stabilization of nano-FeS using loading tends to rely more on adsorption than reduction, and most of the more popular stabilizers are synthetic natural organics with slow natural degradation. Therefore, on the basis of existing preparation methods, there is an urgent need to explore a synthesis method of FeS nanoparticles with a simple preparation process, good economic efficiency and can be prepared with stronger reactivity and better removal performance for Cr(VI) and total chromium.

When an ultrasonic wave travels in a liquid medium, the cavitation effect can destroy objects and reduce their cluster. The use of ultrasound in the order to prepare nanomaterials can significantly prevent the growth and nucleation rate of crystals^24^. The prepared materials are finer and more uniform^25,26^. Although the advent of ultrasound has opened up a new field for the preparation of nanomaterials, the variety of nanomaterials prepared by sonochemical methods is still limited, and there are few reports on the preparation of nano FeS using ultrasound-assisted methods.

The aim of this study is to reduce the agglomeration problem of nano FeS during the treatment of acidic chromium-containing wastewater. The nano FeS prepared by ultrasonic precipitation method was utilized to find out the optimum conditions for the preparation of nano FeS by one-factor (reaction temperature, Fe/S molar ratio and FeSO_4_ dropping flow rate) experiments combined with the response surface method (RSM), using the Cr(VI) removal rate as the evaluation index. In addition, the optimized FeS nanomaterials and their immobilized particles were used in dynamic operation experiments under a continuous reactor to simulate the actual process flow of treating chromium-containing wastewater. The Cr(VI) and total chromium removal effects, TFe and COD releases and effluent turbidity, combined with the characterization of the particles before and after the reaction by SEM and XRD, reveal the removal mechanism and pollutant releases in the dynamic reaction, and provide theoretical references for practical applications.

## Materials and methods

### Experimental materials

FeSO_4_·7H_2_O, Na_2_S·9H_2_O were purchased from Damao Chemical Reagent Co. Ltd. (Tianjin, China). K_2_Cr_2_O_7_ were purchased from Quanrui Reagent Co. Ltd. (Liaoning, China). To obtain Cr(VI) stock solution at a concentration of 100 mg/L, the dried K_2_Cr_2_O_7_ of 0.2829 g was mixed in 1 L of distilled water. The initial pH of the solution was adjusted using 1% HCl and NaOH.

### Preparation of nano-FeS

3.6072 g of Na_2_S·9H_2_O was taken that weighted equivalent to the amount of FeSO_4_·7H_2_O. According to a certain n(Fe)/n(S), dissolve both in 100 mL of deionized water. Place the conical flask having the Na_2_S solution into the ultrasonic cleaning machine. The FeSO_4_ solution was added dropwise into the Na_2_S solution with the help of a peristaltic pump. After ultrasonic treatment, was placed in a centrifuge at rpm of 4000 r/min for 10 min. After that, it was washed with deionized water for 3 times. A vacuum drying oven was used for the drying of it.

### Preparation of nano-FeS immobilized particles

The mass fraction of 9% polyvinyl alcohol and 1% sodium alginate was weighted and dissolved in distilled water. It was sealed and soaked for 24 h. It was then placed in the water bath at a constant temperature of 90 °C for 1.5 h. While in a water bath it was continuously stirred until no bubbles were formed. It was again sealed and cooled to room temperature of 25 °C. 5% nano-FeS weighed into the gel and stirred thoroughly. A peristaltic pump was used for sucking the nano-FeS gel, which was then dropped directly into a 2% CaCl_2_ saturated boric acid solution (pH = 6). Stirring was continually done it. Particles were taken out after 4 h and washed thrice with 0.9% NaCl solution.

### Experimental procedures

This paper consists of three parts:

(1) Static experiment: One gram of nanoFeS centrifugal precipitate was weighed and added to 250 mL of acidic chromium-containing wastewater at pH 4 with an initial Cr(VI) mass concentration of 100 mg/L. The reaction temperatures (5, 15, 25, 35, 45, 55, and 65 °C, 1#~7# systems), Fe/S molar ratios (0.5, 1, 1.5, 2, 2.5, 3, and 3.5. 1#–7# systems) and FeSO_4_ dropping flow rate (0.22, 0.44, 0.66, 0.88 and 1.1 mL/s, 1#–5# systems) on the treatment effect of Cr(VI). The samples were stirred at 600 r/min for 1 h with a magnetic stirrer and taken at 10 min intervals. The concentration of Cr(VI) was measured after passing through a 0.22 μm microporous membrane. Two sets of parallel experiments were also set up.

(2) Optimization of nano-FeS preparation method: Based on the single-sided experiments The Box-Behnken model was used to design the RSM optimization experiments. Design-Expert 8.0.6 software was used for data analysis, and the experimental factor codes and levels are shown in Table 1.

**Table 1:** Ultrasonic precipitation method RSM design factor horizontal coding table.

Factor	Code	Coding level		
		− 1	0	1
Reaction temperature (°C)	A_1_	10	15	20
Fe/S molar ratio	B_1_	2.5	3.0	3.5
Dropping flow rate (mL/s)	C_1_	0.22	0.44	0.66

The relationship between the variation of the response value and the factor is shown in the following equation.

1 $$   {\text{Y}} = \beta _{0}  + \mathop \sum \limits_{{{\text{i}} = 1}}^{{\text{k}}} \beta _{{\text{i}}} {\text{X}}_{{\text{i}}}  + \mathop \sum \limits_{{{\text{i}} = 1}}^{{\text{k}}} \beta _{{{\text{ii}}}} {\text{X}}_{{\text{i}}}^{2}  + \mathop \sum \limits_{{{\text{i}} < j}} \beta _{{{\text{ij}}}} {\text{X}}_{{\text{i}}} {\text{X}}_{{\text{j}}}  + \varepsilon $$

In the formula: *Y* represents the system response value; *β*_*0*_ represents the offset term offset coefficient; *β*_*i*_ represents the linear offset coefficient; *β*_*ii*_ represents the second-order offset coefficient; *β*_*ij*_ represents the interaction coefficient; *X*_*i*_, *X*_*j*_, *X*_*i*_*X*_*j*_ is the level values of each factor to analyze the main effect and interaction effect of each factor.

**Figure 1:**
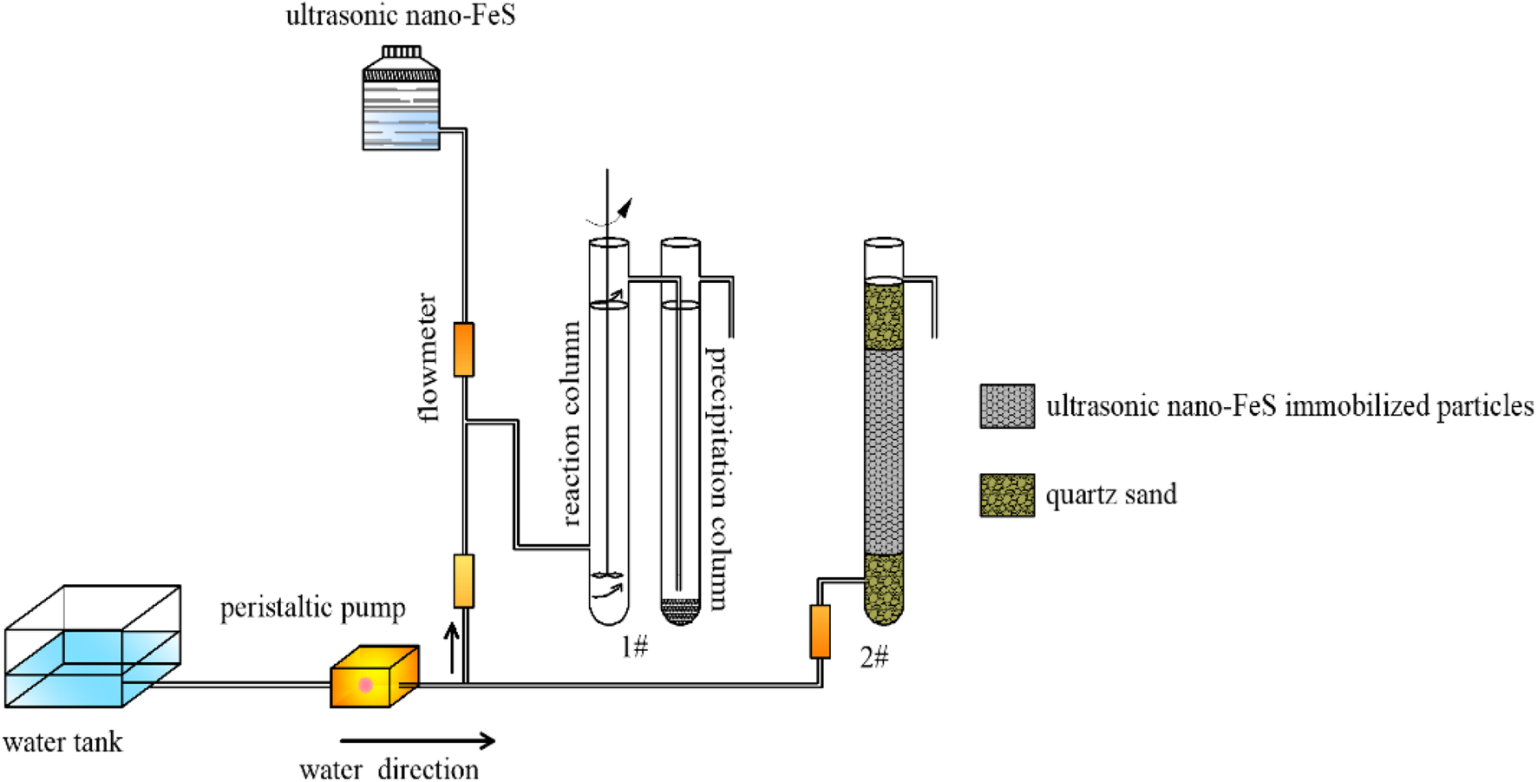
Dynamic test device system.

(3) Dynamic experiments. Two plexiglas columns with an inner diameter of 55 mm and a height of 500 mm were used as reaction vessels, and the experimental setup is shown in Fig. 1. 1# is a dynamic column of ultrasonic nano-FeS stirred at 300 rpm with a solid–liquid ratio of 1:200. 2# is a dynamic column of ultrasonic nano-FeS immobilized particles. The bottom of the reactor is a 50 mm layer of quartz sand, the middle is 350 mm immobilized particles, and the upper is about 50 mm quartz sand layer. The concentration of Cr(VI) in the influent sample was 50 mg/L (pH = 4). The device was operated for 8 days in a "bottom-in, top-out" continuous injection mode with an injection flow rate set at 0.5 mL/min and sampling every 12 h.

### Analysis methods

The concentration of Cr(VI) was determined at a wavelength of 540 nm using a UV–Visible spectrophotometer (Q/TBCR1, Xinmao Instruments Co., Ltd., Shanghai, China) according to the standard of the Chinese Ministry of Environmental Protection (GB/T 7467-1987). Total chromium (TCr) was determined using the potassium permanganate oxidation-diphenylcarbonyldihydrazide spectrophotometric method (GB 7466-87). Total iron (TFe) was determined by the colorimetric method of o-phenanthroline (MT/T 368-2005). pH was determined by the electrode method (HJ 1147-2020). turbidity was determined by turbidimeter (HJ 1075-2019). COD was determined by fast-elimination spectrophotometry (HJ/T 399-2007). TEM was measured by JEOL JEM-2100F. Specific surface area and pore size were measured using Mack ASAP 2460. The surface structure of the samples was scanned using a FEI Inspect F50 scanning electron microscope. The physical phase analysis was determined by Rigaku Smartlab9 X-ray diffractometer.

## Results and discussion

### Static experiment

(1) Temperature effects on the preparation of nano-FeS

**Figure 2:**
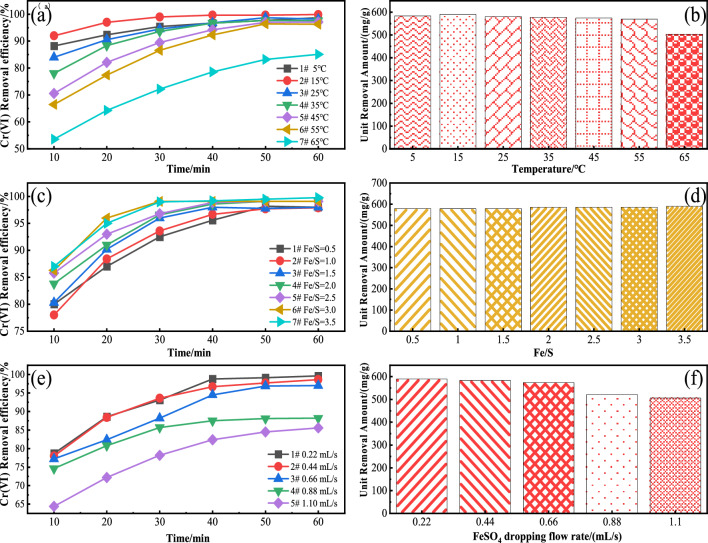
**(a)**-**(b)** Removal efficiency of Cr(VI) by nano-FeS prepared under different reaction temperatures; **(c)**-**(d)** Removal efficiency of Cr(VI) by nano-FeS prepared under different Fe/S molar ratio conditions; **(e)**-**(f)** Removal efficiency of Cr(VI) by nano-FeS prepared under different flow rates.

Figure 2a and b shows the effect of reaction temperature on the treatment of acidic wastewater containing Cr(VI) with FeSO_4_ drop adding flow rate of 0.44 mL/s and Fe/S molar ratio of 3. At the reaction equilibrium, the removal rate and unit removal rate of Cr(VI) were the maximum at 15 °C with 99.9% and 590.86 mg/g, respectively. Temperature has a great influence on the generation and growth of grains, when the amount of solute is certain, the increase in temperature leads to a decrease in the supersaturation of the solution, when the temperature is very low, although the supersaturation is large, but the energy of the solute molecules is very low, and the inter-ionic reaction is inactive, which leads to a very small rate of grain generation; with the increase of the temperature, the rate of its generation is getting larger and larger, and stable and uniform FeS crystals with a uniform particle size are obtained^27^. As the temperature continues to rise, the degree of supersaturation decreases, the kinetic energy of molecules increases rapidly, which is not conducive to the formation of stable grains; the degree of Brownian motion of molecules is intensified, increasing the probability of intermolecular collisions, which makes it easier for nanoparticles to agglomerate with each other through intermolecular forces, inter-particle quantum tunneling effects, charge transfer and interfacial atomic mutual coupling, and the occurrence of interactions and solid-phase reactions^28^. When the temperature is less than 15 °C lower temperatures are favorable for grain generation, unfavorable for grain growth, and inhibit the oxidation of FeS, making its removal more effective. Some studies have shown that the temperature increases by 20 °C, the precipitation salt crystal particles increase by 10% to 20%^27^, so when the temperature is greater than 15 °C, the larger the temperature, the larger the crystal particle size than the surface area and surface activity decreases and the molecular energy increases the oxidation easily, affecting its removal of Cr (VI). In summary, different reaction temperature preparation of nano-FeS has a great influence on the removal rate, removal rate and unit removal amount of Cr(VI), the reaction temperature conditions of T = 15 °C were selected for response surface optimization. In summary, a reaction temperature of 15 °C was chosen to optimize the RSM.

(2) Fe/S molar ratio effects on the preparation of nano-FeS

Figure 2c and d shows the effect of nano-FeS prepared at different Fe/S molar ratios on the removal rate and unit removal of Cr(VI) from acidic wastewater. Both the removal rate and unit removal of Cr(VI) increased with the increase of Fe/S molar ratio. When the Fe/S molar ratio was 3.5, the removal rate and unit removal rate of Cr(VI) were the maximum of 99.8% and 590.27 mg/g, respectively. The unit removal amount of 1#–3# systems with Fe/S = 0.5–1.5 is the same, the unit removal amount of 4#–6# systems with Fe/S = 2.0–3.0 is the same, and the unit removal amount of 4#–6# systems is larger than that of 1#–3# systems. Because the solution concentration has a great influence on the nucleation and growth of crystals, which has a greater influence on the rate of grain generation, when the concentration of Fe^2+^ ions constituting FeS crystals in the solution increases it is more favorable to the increase in the number of grains. When the solution concentration is higher, the faster the rate of grain generation, the generation of more and smaller grains, so the growth of grains is slow, so that it is too late to grow^27^. It makes the nano-FeS crystals with smaller particle size, which can provide larger collision area and more active sites with stronger specific surface area and activity^29^, thus effectively promoting the rapid occurrence of the reaction. In summary, the preparation of nano FeS with different Fe/S molar ratios has a great influence on the removal rate and removal rate of Cr(VI), and considering that larger Fe/S molar ratios result in the waste of resources, the reaction condition with Fe/S molar ratio of 3 was selected as the parameter for optimization experiments.

(3) Different FeSO_4_ dropping flow rates on the preparation of nano-FeS

Figure 2e and f depicts the effect of nano-FeS prepared with different FeSO_4_ dropping flow rates on the removal rate and unit removal amount of Cr(VI) in acidic wastewater. The removal rate and unit removal of Cr(VI) decreased with the increase of FeSO_4_ drop addition flow rate. When the FeSO_4_ drop addition flow rate was 0.22 mL/s, the removal rate and unit removal of Cr(VI) were the highest 99.6% and 589.09 mg/g, respectively. the collision of Fe^2+^ and S^2−^ in the solution produced nano-FeS crystals, in which Fe^2+^ was involved in the nucleation and growth process. When the flow rate of FeSO_4_ was low, Fe^2+^ was involved in both nucleation and growth processes. With the depletion of Fe^2+^, the nucleation process gradually stops, which hinders the growth of the crystals^30^, and smaller FeS nanoparticles with higher surface energy are generated, which can rapidly consume contaminants. When the dropwise flow rate of the Fe^2+^ solution is small, the amount of reactants is limited, which also controls the overly rapid crystal growth and agglomeration between crystals due to the generation of crystal bridges. From Weiman's theory of supersaturated nucleation and growth rate, the nucleation and growth rates are very low at this time^31^. When the drop addition flow rate is large, Fe^2+^ is sufficient to supply the growth of both small and large size grains, resulting in an increase in the size of FeS nanoparticles, a decrease in specific surface area, and a decrease in surface free energy. The removal rate and unit removal amount of Cr(VI) by the nano-FeS prepared when the flow rate of FeSO_4_ was 0.44 mL/s was similar to that at 0.22 mL/s, and the preparation time could also be shortened. Therefore, the FeSO_4_ dropping flow rates of 0.44 mL/s was chosen as the optimized parameter.

### Analysis of RSM

Single factor experiments have limitations because they do not allow for analysis and optimization of successive points and do not reflect factor interactions. Therefore, based on the one-factor experiment, the BBD model^32^ was used for a three-factor, three-level design with reaction temperature, Fe/S molar ratio and FeSO_4_ dropping flow rate initially determined in the one-factor experiment as the central values, and the Cr(VI) removal rate (Y) as the response value, and a response surface experiment was conducted. Response surface experiments were carried out. According to the single factor test results, the RSM test was carried out, and the test results are shown in Table 2.

**Table 2:** The experimental results of RSM.

No	Variable						
	Actual value			Encoded value			Cr(VI) response
	A_1_	B_1_	C_1_	A_1_	B_1_	C_1_	/%
1	10	2.5	0.44	− 1	− 1	0	75.8
2	20	2.5	0.44	1	− 1	0	62.0
3	10	3.5	0.44	− 1	1	0	83.5
4	20	3.5	0.44	1	1	0	73.4
5	10	3.0	0.22	− 1	0	− 1	78.7
6	20	3.0	0.22	1	0	− 1	69.0
7	10	3.0	0.66	− 1	0	1	76.4
8	20	3.0	0.66	1	0	1	62.5
9	15	2.5	0.22	0	− 1	− 1	78.7
10	15	3.5	0.22	0	1	− 1	81.6
11	15	2.5	0.66	0	− 1	1	64.4
12	15	3.5	0.66	0	1	1	80.5
13	15	3.0	0.44	0	0	0	79.0
14	15	3.0	0.44	0	0	0	79.1
15	15	3.0	0.44	0	0	0	78.5
16	15	3.0	0.44	0	0	0	79.6
17	15	3.0	0.44	0	0	0	78.4

According to the results in Table 2, through Box-Behnken experimental calculation, the quadratic polynomial regression model of the preparation of nano-FeS treated Cr(VI) under different factors is obtained as follows:

2 $${\text{Cr}}\left({\text{VI}}\right)\text{Removal Rate}\left(\text{\%}\right)\text{=78.92-5.94}{\text{A}}_{1}\text{+4.76}{\text{B}}_{1}\text{-3.02}{\text{C}}_{1}\text{+0.92}{\text{A}}_{1}{{\text{B}}}_{1}\text{-1.05}{\text{A}}_{1}{{\text{C}}}_{1}\text{+3.3}{\text{B}}_{1}{{\text{C}}}_{1}\text{-4.95}{{\text{A}}_{1}}^{2}\text{-0.3}{{\text{B}}_{1}}^{2}\text{-2.32}{{\text{C}}_{1}}^{2}$$

Analysis of variance was performed on the second-order model, as shown in Table 3.

**Table 3:** Analysis of variance of regression model for Cr(VI) removal rate.

Source	Sum of square	Degrees of freedom	Mean square	F	*P*	Salience
Model	721.40	9	80.16	87.59	< 0.0001	Significant
A_1_	282.03	1	282.03	308.21	< 0.0001	***
B_1_	73.20	1	181.45	198.29	< 0.0001	***
C_1_	25.60	1	73.20	80.00	< 0.0001	***
A_1_B_1_	3.42	1	3.42	3.74	0.0944	⊙
A_1_C_1_	4.41	1	4.41	4.82	0.0642	⊙
B_1_C_1_	43.56	1	43.56	47.60	0.0002	***
A_1_^2^	103.06	1	103.06	112.63	< 0.0001	***
B_1_^2^	0.37	1	0.37	0.41	0.5437	⊙
C_1_^2^	22.71	1	22.71	24.82	0.0016	**
Residual tern	6.41	7	0.92			
Lack of fit	5.46	3	1.82	7.68	0.0390	
Pure error	0.95	4	0.24			
Sum	727.80	16				
Coefficient of variation	1.27%	Adeq precision	29.731	R^2^ = 0.9912	R^2^_Adj_ = 0.9799	R^2^_Pred_ = 0.8780

***, *P* < 0.001, extremely significant; **, *P* < 0.01, highly significant; *, *P* < 0.05, significant; ⊙, not significant.

The RSM test results are shown in Table 2. The multivariate correlation coefficient R_2_ = 0.9912 , which has a low degree of distortion. The revised complex correlation coefficient R^2^_Adj_ = 0.9799 and it has a good degree of fit and regression. The F value of the equation is 87.59, and the P value is less than 0.0001 (0.05), indicating that the calculation model has reached a significant level and can be used to replace the actual point of the test for result analysis, as shown in Table 2. The F values for reaction temperature, Fe/S molar ratio, and FeSO_4_ dripping flow rate were 308.21,198.29, and 80, respectively, and the P values for all three factors were all less than 0.0001. The three factors have a significant influence on the removal rate of Cr(VI), in the following order: reaction temperature > Fe/S molar ratio > FeSO_4_ dripping flow rate.

**Figure 3:**
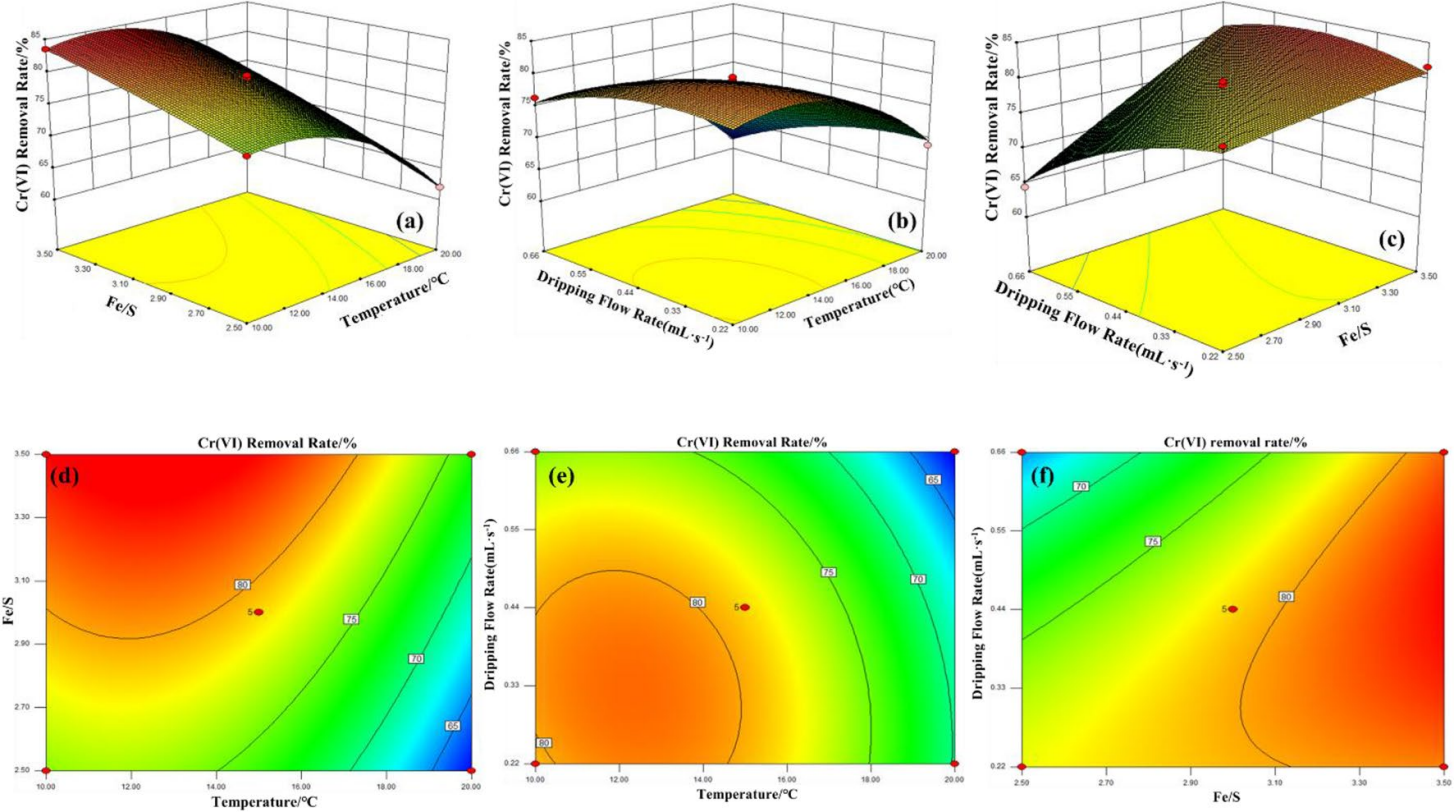
Contour map and RSM diagram of the effect of nano-FeS on the removal rate of Cr(VI) by ultrasonic precipitation.

The RSM and contour maps are depicted in Fig. 3a–c and d–f. The RSM diagram in Fig. 3a showed the effect of reaction temperature and Fe/S molar ratio on the rate of removal of Cr(VI) by the prepared nano-FeS when the FeSO_4_ dripping flow rate was 0.44 mL/s. When the reaction temperature was constant, the removal rate of Cr(VI) increases with increasing Fe/S molar ratio. When the Fe/S molar ratio was constant, the removal rate of Cr(VI) increases first and then decreases with increasing reaction temperature. The interaction between reaction temperature and Fe/S molar ratio has a P value of 0.0944 (> 0.05) representing that there was non-significant interaction between them. When the Fe/S molar ratio is 3, the RSM diagram of the effect of reaction temperature and FeSO_4_ dripping flow rate on the removal rate of Cr(VI) by the prepared nano-FeS is shown in Fig. 3b. Lowering the FeSO_4_ solution's dropping flow rate and reaction temperature could promote the removal of Cr(VI) by the prepared crystals of nano-FeS. The interaction between reaction temperature and FeSO_4_ dripping flow rate has a P value of 0.0642 (> 0.05), revealing that there was an interaction but that it was not significant. The RSM diagram in Fig. 3c depicted the effect of Fe/S molar ratio and FeSO_4_ dropping flow rate on the Cr(VI) removal rate of prepared nano-FeS at 15 °C. The chart revealed that when the Fe/S molar ratio was constant, the removal rate of Cr(VI) decreases with increasing FeSO_4_ dripping flow rate. When the Fe/S molar ratio was constant, the removal rate of Cr(VI) increases with increasing FeSO_4_ dripping flow rate. The interaction between Fe/S molar ratio and FeSO_4_ dropping flow P value was 0.0002 which is less than 0.05 thus indicating that the association was fairly significant. According to the RSM graph and regression model results, the removal rate of Cr(VI) is 84.73% when Fe/S = 3.5, the reaction temperature was 12.37 °C and the dropping flow rate was 0.5 mL/s.

According Fig. 3d–f, the contour lines of the three graphs all showed a very clear ellipse, thus indicating that the interaction of these three factors was very significant. Combined with the regression model variance, the interaction strength of the three factors could be obtained as follows: Fe/S molar ratio and FeSO_4_ dropping flow rate > reaction temperature and FeSO_4_ dropping flow rate > reaction temperature and Fe/S molar ratio respectively.

### TEM analysis and N_2_ adsorption test analysis

**Figure 4:**
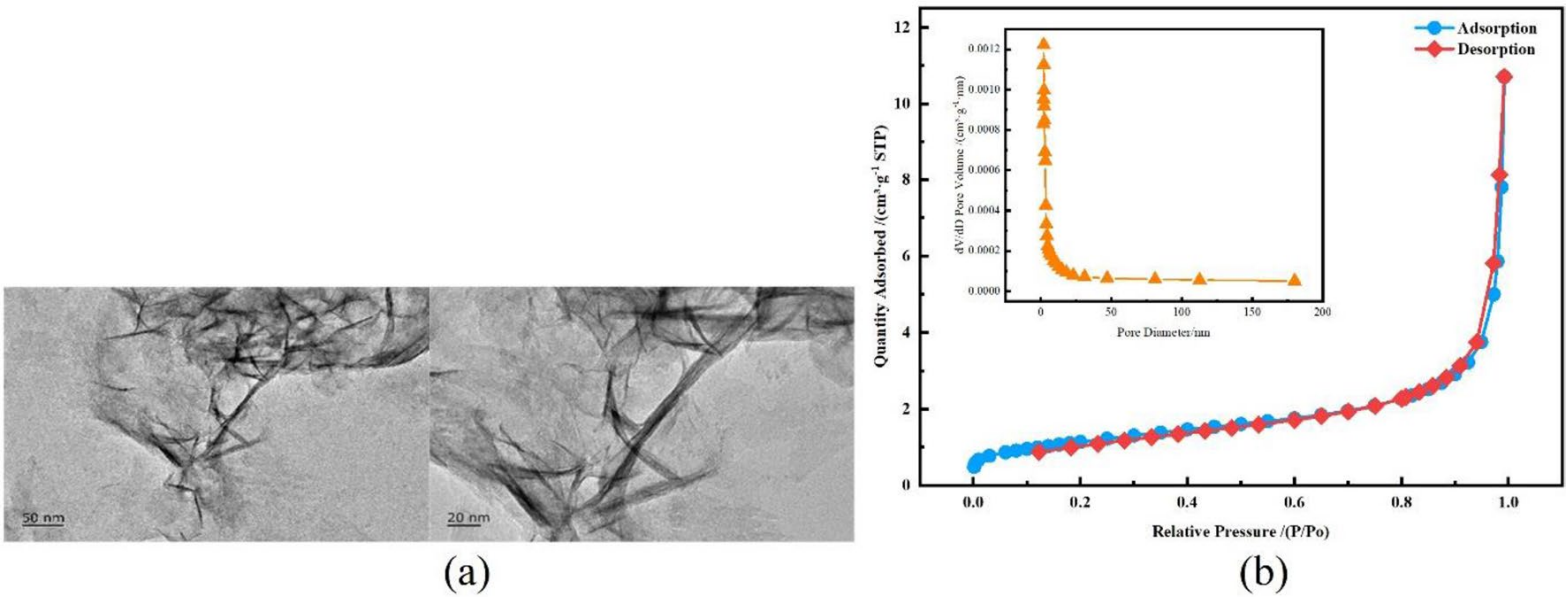
**(a)**TEM images of nano FeS;**(b)** Nano FeS materials nitrogen adsorption–desorption isotherms and Pore-aperture differential distribution curves.

Nano-FeS was mainly needle-shaped and whisker-shaped. It has a length of 40–80 nm and a width of 5–8 nm, which was nano-scale FeS (Fig. 4a). The morphology of a crystal was comparable to that of nano-FeS that were prepared by the homogeneous precipitation method by Chen et al^33^. It indicated that nano-FeS could be prepared by ultrasonic precipitation method and nano-FeS is relatively dispersed ^34^.

Figure 4b shows the N_2_ adsorption–desorption isothermal curve of FeS nanoparticles, and the inset shows the BJH pore size distribution curve.

The results calculated according to the BET isothermal adsorption curve equation and the BJH method ^35^ are shown in Table 4. The BET surface area of nano-FeS crystals prepared by ultrasonic precipitation method is 4.1445 m^2^/g, the average pore volume is 0.016883 cm3/g, and the average pore diameter is 5.65023 nm. According to the five types (I–V) of isothermal adsorption curves for adsorption of gases on solids summarized by Brunauer et al^36^, the curves belong to type III, in which the interaction force between the gas molecules and the surface of the solid is smaller than that between the gas molecules and the gas molecules, the increase of adsorption amount of this type adsorption curve is not obvious when the relative pressure is small, which indicates the weak force with nitrogen; when the relative pressure is large, the adsorption amount at the high pressure end is large, and the curve goes up and the adsorption amount increases significantly for the pores generated by particle accumulation^17^. In summary, the adsorption curve is consistent with the Freundlich isothermal adsorption curve. From the BJH inset, it can be seen that there is no concentrated distribution of pore channels in the mesoporous range of nano-FeS, and the average pore size of nano-FeS was calculated, indicating that there is a certain amount of micropores in nano-FeS.

**Table 4:** Pore structure parameters of nano-FeS.

Specific surface area (m^2^/g)			Pore volume (cm^3^/g)		Aperture (nm)
BET	Langmuir	t-Plot External	BJH Adsorption	BJH Desorption	BET Adsorption
4.1445	10.5813	4.1660	0.016883	0.017167	5.65023

### The dynamic test of nano-FeS and its immobilized particles in the treatment of acidic chromium-containing wastewater

#### Analysis of Cr(VI) removal effect

**Figure 5:**
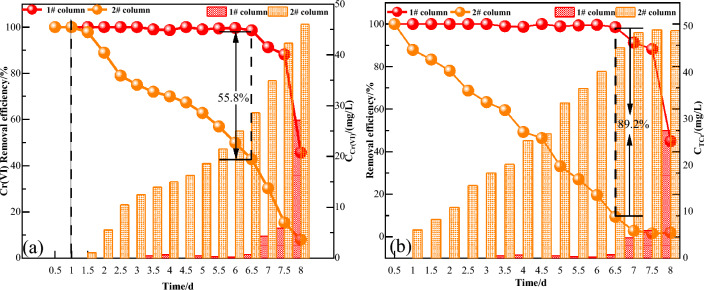
**(a)** Cr (VI) removal effect;**(b)** Total chromium removal effect (solution pH: 4, Cr(VI) initial concentration: 50 mg/L).

Figure 5a shows the removal effect of Cr(VI) by 1# and 2# dynamic columns. The removal rate of Cr(VI) was stable from 99.4 to 100% in 1# dynamic column. After 6.5 days, the removal rate gradually decreased from 98.6 to 45.7%. the removal rate of Cr(VI) in 2# dynamic column decreased from 100 to 8%. The average removal rates of Cr(VI) by the two sets of dynamic columns were 94.97% and 63.51%, respectively. Compared with the immobilized particles, the removal of Cr(VI) from acidic wastewater was much better with FeS nanoparticles. This is because the acidic feed water prompted the precipitation of Fe^2+^ and S^2−^ on the surface of the nano-FeS particles and immobilized particles, which rapidly reduced Cr(VI) to Cr(III), so the removal rate of Cr(VI) in both dynamic columns was 99.5% before 1 d. The nano-FeS particles in the dynamic column #1 continuously precipitated Fe^2+^ and S^2−^ under the acidic environment, and continuously contacted and collided with the CrO_4_^2−^ in the feed water for oxidation. The removal rate of Cr(VI) was high until 6.5 d. After 6.5 d, the nano-FeS was gradually depleted, so that the removal rate of Cr(VI) in column #1 started to decrease.The nano-FeS on the surface of the immobilized particles in the #2 dynamic column was gradually depleted and the active sites were reduced^37^, which is consistent with previous studies on iron passivation under neutral and alkaline conditions^38^, and a passivation film was gradually formed to hinder the electron transfer between the surface of the immobilized particles and Cr(VI), resulting in a greater resistance to the diffusive transport of CrO_4_^2−^ to deeper layers of the particles, and CrO_4_^2−^ was not completely The CrO_4_^2−^ was not completely reduced before it flowed out with the effluent. Therefore, the reduction rate of Cr(VI) in 2# dynamic column continued to decrease after 1 d. Wu et al^17^ stabilized FeS nanoparticles with sodium alginate and achieved 100% removal of Cr(VI) in 160 min, which was 6% higher than the results of this experiment due to the low initial concentration of Cr(VI). Chen et al^39^ modified zero-valent iron nanoparticles with porous boron nitride of high specific surface area and obtained 98.1% removal of Cr(VI).Ye et al^40^ used modified nano zero-valent iron to remove 60% of Cr(VI) in 1440 min. The removal of Cr(VI) by FeS nanoparticles prepared in the present work was significantly better than that of modified nano zero-valent iron compared to the past studies.

#### Analysis of total chromium removal effect

The removal effects of 1# and 2# dynamic columns on total chromium are shown in Fig. 5b. By comparing the two sets of dynamic experimental columns, the average removal rates of total Cr were calculated to be 94.93% and 45.76%. The reason was found to be that the 1# dynamic column could make full contact between nano-FeS and CrO_4_^2−^ through mechanical hydraulic stirring to enhance the reduction effect. As H^+^ was continuously consumed leading to the increase of pH, Fe^2+^ and Fe^3+^ were hydrolyzed in water to generate FeOOH, Fe(OH)_2_ and Fe(OH)_3_ precipitated flocs and had obvious adsorption effect on Cr^3+^ in total chromium^41^. Therefore, the removal rate of total chromium in 1# dynamic column was always stable above 98.7% in 1–6.5 d. With the gradual consumption of nano-FeS, the removal rate of total chromium gradually decreased from 6.5 d. The 2# dynamic column used immobilization method to immobilize nano-FeS in spherical particles, which greatly reduced the contact with nano-FeS, thus making the redox reaction rate lower than that of the 1# dynamic column. As the reaction proceeds, Fe^2+^ and H^+^ are gradually consumed. A passivation film adheres to the surface of the particles in neutral or alkaline solutions^38^. S^2−^ also forms a form of Cr_2_S_3_ precipitation with free Cr^3+^, which blocks the pores inside the particles and affects the rate of material transfer between immobilized particles, leading to poor removal of Cr(III) in the later stages of the reaction, resulting in a continuous decrease in the total chromium removal rate of the 2# dynamic column. Mantovani et al.^42^ used microalgae-modified nano-zero-valent iron to remove 12.4% of total chromium in 54 h. Gajaraj et al.^43^ removed 66.2% and 56.7% of total chromium from wastewater after 50 days with the help of microbial electrolytic cell and microbial fuel cell, respectively. Sahinkaya^44^ et al. used elemental sulfur as an electron acceptor and through this technique, the removal rate of total chromium reached 85% after 250 d. This result was lower than the current experiment and this might be due to the halting of the production of HS gas ^45^ that could lower Cr(VI). As the reaction temperature increased it was found that the total Cr removal rate gets decreased. In comparison with the above studies, the nano FeS materials prepared in this study are more beneficial in the confiscation of total chromium.

#### Analysis of TFe, COD release, pH, and turbidity changes

**Figure 6:**
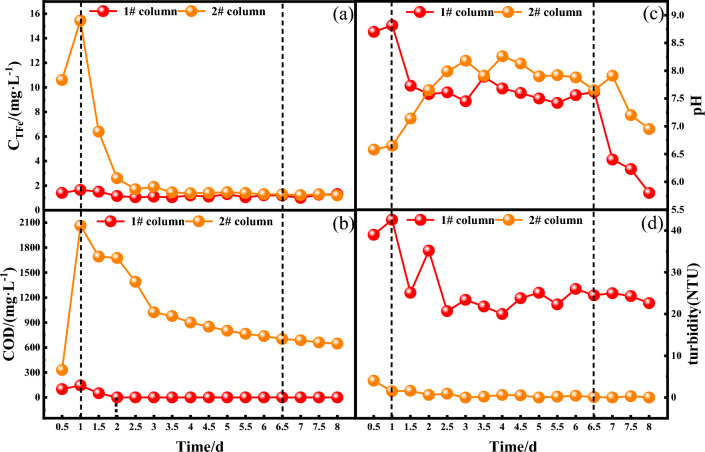
**(a)**TFe release, **(b)** COD release, **(c)** change of pH, and **(d)** turbidity analysis (solution initial pH: 4, Cr(VI) initial concentration: 50 mg/L^)^.

Figure 6 shows the changes of TFe release, COD release, pH and turbidity in the dynamic columns 1# and 2#. According to Fig. 6a, the release of TFe in the #1# dynamic column was always low with an average release of 1.22 mg/L. The release of TFe in the 2# dynamic column increased sharply from the beginning of the reaction, reaching a peak of 15.45 mg/L at 1 d, and then decreased gradually. As can be seen from Fig. 6b, the COD release from dynamic columns 1# and 2# reached a peak of 145 mg/L and 2064 mg/L at 1 d of the reaction, respectively, and then started to decrease, and the release from dynamic column 1# decreased to 0 mg/L at 2 d. It can be seen that the release of TFe and COD from dynamic columns with immobilized particles was significantly higher than that from dynamic columns without immobilized FeS nanoparticles. This is due to the lower pH of the influent water, the Fe^2+^ and S^2−^ precipitated rapidly with Cr(VI) in the redox reaction by the FeS nanoparticles in the 1# dynamic column, and the generated Fe(OH)_3_, FeOOH and Cr_2_S_3_ precipitated flocs, so that the total amount of reduced Fe^2+^ and S^2−^ in the solution decreased rapidly, and thus the TFe content and COD values in the solution of the 1# dynamic column were always lower. In the 2# dynamic column, Fe^2+^ precipitated from the nano-FeS on the surface of immobilized particles was oxidized to Fe^3+^ through the reaction with Cr(VI), which led to a significant increase in the release of TFe. At the same time, the organic matter in the immobilized particles prepared with polyvinyl alcohol and sodium alginate was dissolved and diffused into the solution, resulting in a higher COD value in the effluent. With the gradual depletion of surface FeS, the pores inside the immobilized particles were blocked by precipitates, which increased the release of Fe^2+^ and organic matter from the deeper layers of the particles and hindered the efficient redox reaction^4,46^, and the continuous influent water also played a diluting role in the concentration of TFe and the amount of COD in the dynamic column, leading to a continuous decrease in the TFe and COD values at the later stages of the reaction.

As can be seen in Fig. 6c, the pH of both dynamic columns increased rapidly to 8.7 and 6.58 after the start of the reaction. pH of column 1# had a maximum of 8.82 at 1 d and a minimum of 5.8 at 8 d. The pH of column 2# had a maximum of 8.26 at 4 d, fluctuated from 7.65 to 8.26 from 3 to 7 d, and decreased again to 6.95 from the 7th day of the reaction. The pH enhancement ability of 2# dynamic column with immobilization was stronger than that of 1# dynamic column. At the beginning of the reaction, the uncured nanoscale FeS in the 1# dynamic column reacted more rapidly and rapidly consumed a large amount of H^+^, resulting in a rapid increase in pH. The pH in the middle of the reaction is weakly alkaline due to the precipitation of Fe^2+^ and S^2−^ from the nano-FeS under acidic conditions while consuming H^+^, and the combination of S^2−^ with H^+^ in water to form HS^−^ or H_2_S, which leaves the solution as a gas, resulting in a continuous decrease in the H^+^ concentration in the solution ^47^. As the nano-FeS was consumed in the 2 dynamic columns, not enough Fe^2+^ and S^2−^ could be precipitated to combine with H^+^ in the feed water, so the pH of the effluent from the two sets of dynamic columns continued to decrease in the later stage.

In the Fig. 6d, it could be seen that the turbidity of the effluent of the immobilized particle dynamic column was lesser as compared to the nanoparticle dynamic column. This was because the greater flocculent precipitates produced during the reaction of the 1# dynamic column were progressively disseminated into minor flocculent precipitates during the stirring procedure, which could not be removed in the precipitation column. At the start of the reaction in 2# dynamic column, Fe^2+^ precipitated from the FeS on the surface of the particles in the acidic wastewater was oxidized to Fe^3+^ and a small amount of Fe(OH)_3_ was formed. As the reaction remains, Fe(OH)_3_ precipitates in the solidified particles, so the turbidity of the effluent from the 2# dynamic column was always very low.

### SEM and XRD analysis

**Figure 7:**
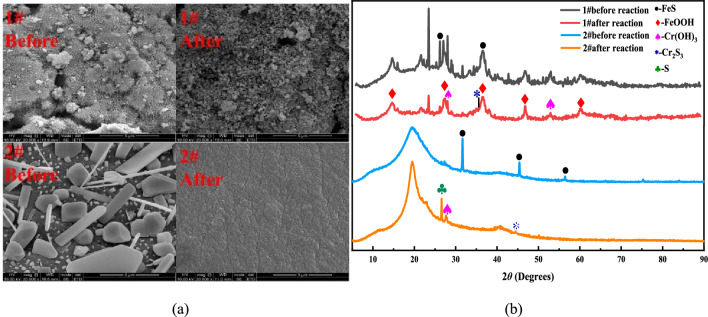
SEM images of nano FeS before and after the reaction (**a**); XRD images of nano FeS before and after the reaction (**b**).

It can be seen from Fig. 7a that the surface of FeS nanoparticles has transformed from short rods to spherical before and after the reaction. There were also found obvious deposits on its surface. This was because FeS was transformed into Fe(OH)_2_ and FeOOH, where the Cr^3+^ in the form of Cr(OH)_3_ and Cr_2_S_3_ deposited on. The immobilized particles were evenly distributed in rod-like and flake-like shapes and had an appropriate size. The original FeS crystals on the particle surface vanished after the reaction and simultaneously the internal substances of the immobilized particles also took part in the reaction and get consumed. The surface was comparatively rough, and small pores were evident. This represented that there were deposits on the surface of the particles. Some ions could enter the interior of the particles complementing the surface holes reaction.

It can be seen from Fig. 7b that the iron element happened in the form of FeS formerly the reaction of the 1# dynamic column, which was principally amorphous and microcrystalline. This was regular with the SEM photo in Fig. 7a. Later in the reaction, new distinctive stages Cr(OH)_3_ and FeOOH appeared in the particles. This indicated that FeS has undergone a redox reaction with Cr(VI), and Cr(VI) was reduced to Cr^3+^. As the H^+^ in the reaction solution was consumed and the pH upsurged, Cr^3+^ and Fe^2+^ generated Cr(OH)_3_ and FeOOH. Part of Cr^3+^ and S^2−^ co-precipitated to form Cr_2_S_3_ precipitates. Before the 2# dynamic column reaction, FeS appeared in the form of FeS^2+^ and there were distinctive diffraction crests of other substances deprived of oxidation. This scenario represented that the immobilized particles could well coat the nano-FeS to avoid being oxidized by air. A novel distinctive stage S appeared in the particles afterward the reaction. This was because the S^2−^ precipitated from nano-FeS in an acidic environment reduced the Cr(VI) to Cr^3+^. This S^2−^ was then oxidized into elemental sulfur, which was captured in the pores of particles. Simultaneously, one part of Cr^3+^ and ionized Fe^2+^ generated Cr(OH)_3_ and FeOOH precipitates respectively under a neutral environment. Another part of Cr^3+^ and S^2−^ co-precipitated to form Cr_2_S_3_ precipitates.

### Mechanism analysis

**Figure 8:**
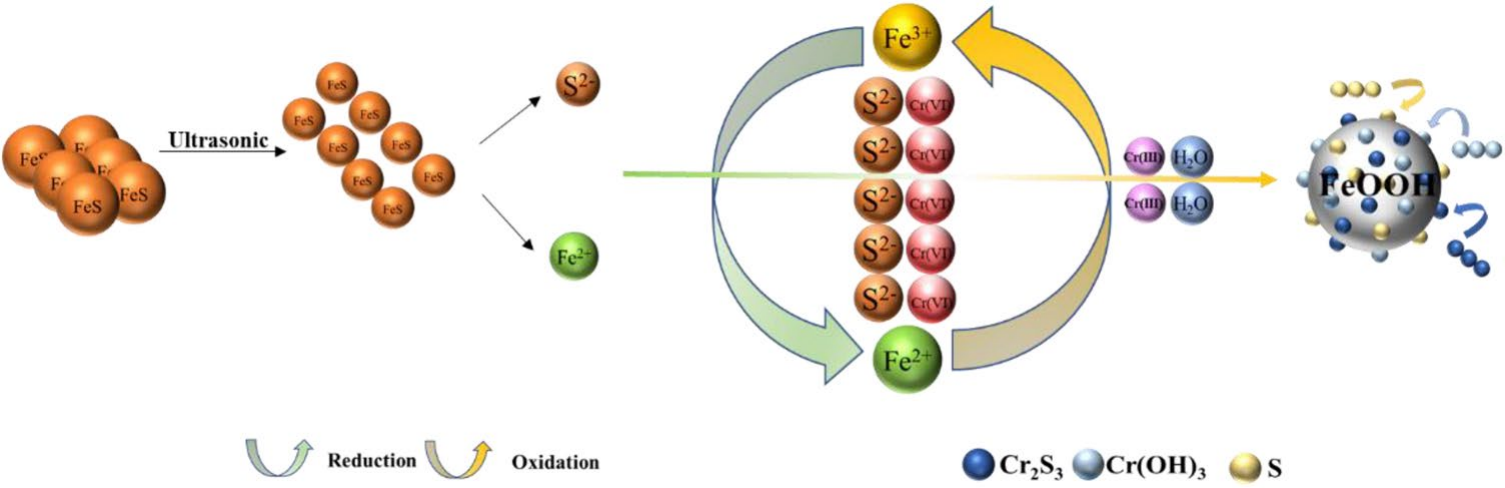
Mechanism of Cr(VI) reduction by FeS nanoparticles.

Based on the results of material characterization and dynamic experiments, the reaction mechanism is shown in Fig. 8. The potential mechanism can be described as follows:

Ultrasound can crush and disperse FeS crystals, thus making the crystal particle size uniform and effectively avoiding crystal agglomeration^48^. The nano-FeS prepared by ultrasonic precipitation method has smaller particle size and higher porosity, which can adsorb Cr(VI) from the solution. In addition FeS nanoparticles prepared by ultrasonication have a large specific surface area and can ionize more Fe^2+^ and S^2−^ with reducing properties. These Fe^2+^ and S^2−^ rapidly undergo redox reactions with Cr(VI) to form Fe^3+^ and Cr(III), the reaction equation is given in Eqs. (3–4)^49^. Some of the S^2−^ that did not react with Cr(VI) may reduce Fe^3+^ to Fe^2+^, forming a small iron cycle that diminishes as the ion concentration decreases. The reaction equation is given in Eqs. (7). As the reduction reaction proceeds, H^+^ is consumed in large amounts, raising the pH of the solution, and Cr_2_S_3_, Cr(OH)_3_ and FeOOH complexes precipitate and attach to the surface of FeS nanoparticles, with the reaction equation shown in Eqs. (5–6)^50^. According to the analysis of SEM and XRD results, the reduction reaction plays a dominant role in the removal of Cr(VI) by nano-FeS.

3 $$3{{\text{Fe}}}^{2+}+{{\text{HCrO}}}_{4}^{-}+7{{\text{H}}}^{+}\to 3{{\text{Fe}}}^{3+}+{{\text{Cr}}}^{3+}+4{{\text{H}}}_{2}{\text{O}}$$

4 $$3{{\text{S}}}^{2-}+2{{\text{HCrO}}}_{4}^{-}+14{{\text{H}}}^{+}\to 3{\text{S}}+2{{\text{Cr}}}^{3+}+8{{\text{H}}}_{2}{\text{O}}$$

5 $${{\text{Cr}}}^{3+}+3{{\text{H}}}_{2}{\text{O}}\to {\text{Cr}}({\text{OH}}{)}_{3}+3{{\text{H}}}^{+}$$

6 $${{\text{xCr}}}^{3+}+(1-{\text{x}}){{\text{Fe}}}^{3+}+3{{\text{H}}}_{2}{\text{O}}\to (3{{\text{Cr}}}_{{\text{x}}}{{\text{Fe}}}_{1-{\text{x}}})({\text{OH}}{)}_{3\left({\text{s}}\right)}+3{{\text{H}}}^{+}$$

7 $${{\text{Fe}}}^{3+}+{{\text{S}}}^{2-}\to {{\text{Fe}}}^{2+}+{\text{S}}$$

## Conclusion

In this study, the conditions of nano FeS prepared by ultrasonic precipitation method were optimized by single factor experiments. Then dynamic continuous experiments were constructed to simulate the process flow of nano FeS and its immobilized particles for treating actual chromium-containing wastewater, and the following conclusions were obtained.

1. The optimal reaction conditions for the preparation of FeS nanoparticles by ultrasonic precipitation are: reaction temperature of 12 °C, Fe/S molar ratio of 3.5, and dropwise flow rate of 0.5 mL/s. The best prepared FeS nanoparticles were in the form of 40–80 nmd needle and whisker particles with good dispersibility and well-defined mesoporous structure.
2. The order of influence of the three factors is: reaction temperature > Fe/S molar ratio > FeSO_4_ dropwise flow rate. The strength of the interaction was Fe/S molar ratio and FeSO_4_ drop rate > reaction temperature and FeSO_4_ drop rate > reaction temperature and Fe/S molar ratio.
3. The typical removal rates of Cr(VI) and total chromium by nano-FeS and its immobilized particles were 94.97% and 63.51%, 94.93% and 45.76%, respectively. The nano-FeS particles have long-term treatment effect on Cr(VI) and total chromium removal.
4. The nano-FeS prepared by sonoprecipitation ionizes more Fe^2+^ and S^2−^, which can rapidly undergo redox reactions with Cr(VI) to form Fe^3+^ and Cr(III). Another part of S^2−^ can reduce Fe^3+^ to Fe^2+^, forming a small iron cycle and decreasing with increasing ion concentration. complexes such as Cr_2_S_3_, Cr(OH)_3_ and FeOOH are attached to the surface of FeS nanoparticles.

This study demonstrates that nano-FeS optimally prepared by ultrasonic precipitation method has long-term treatment effect on acidic chromium-containing wastewater. It is necessary to further investigate the solid–liquid separation technique after reaction to reduce the turbidity of the effluent.

## Data Availability

The datasets used or analyzed during the current study are available from the corresponding author on reasonable request.
